# Re-evaluation of transcription factor function in tomato fruit development and ripening with CRISPR/Cas9-mutagenesis

**DOI:** 10.1038/s41598-018-38170-6

**Published:** 2019-02-08

**Authors:** Rufang Wang, Eveline Carla da Rocha Tavano, Michiel Lammers, Adriana Pinheiro Martinelli, Gerco C. Angenent, Ruud A. de Maagd

**Affiliations:** 1 Laboratory of Molecular Biology, Wageningen University, Wageningen, The Netherlands; 2 Bioscience, Wageningen Plant Research, Wageningen, The Netherlands; 3 CENA, University of Sao Paulo, Piracicaba, Brazil

## Abstract

Tomato (*Solanum lycopersicum*) is a model for climacteric fleshy fruit ripening studies. Tomato ripening is regulated by multiple transcription factors together with the plant hormone ethylene and their downstream effector genes. Transcription Factors APETALA2a (AP2a), NON-RIPENING (NOR) and FRUITFULL (FUL1/TDR4 and FUL2/MBP7) were reported as master regulators controlling tomato fruit ripening. Their proposed functions were derived from studies of the phenotype of spontaneous mutants or RNAi knock-down lines rather than, as it appears now, actual *null* mutants. To study TF function in tomato fruit ripening in more detail, we used CRISPR/Cas9-mediated mutagenesis to knock out the encoding genes, and phenotypes of these mutants are reported for the first time. While the earlier ripening, orange-ripe phenotype of *ap2a* mutants was confirmed, the *nor null* mutant exhibited a much milder phenotype than the spontaneous *nor* mutant. Additional analyses revealed that the severe phenotype in the spontaneous mutant is caused by a dominant-negative allele. Our approach also provides new insight into the independent and overlapping functions of *FUL1* and *FUL2*. Single and combined *null* alleles of *FUL1* and *FUL2* illustrate that these two genes have partially redundant functions in fruit ripening, but also unveil an additional role for *FUL2* in early fruit development.

## Introduction

Tomato (*Solanum lycopersicum*) produces fleshy fruits, which are climacteric, i.e. displaying a burst in ethylene production during ripening. Its diploid genome, available genome sequence and relative ease of transformation make it the ideal model for studying fleshy fruit development and ripening.

Biochemical and physiological processes during tomato fruit ripening result in changes in texture, colour and flavour. Together with ethylene, transcription factors (TFs) and their downstream effector genes regulate these changes^1^. RIPENING INHIBITOR (RIN), COLORLESS NON-RIPENING (CNR), TOMATO AGAMOUS-LIKE1 (TAGL1), APETALA2a (AP2a), NON-RIPENING (NOR) and FRUITFULL (FUL1 and FUL2) are major TFs regulating tomato fruit ripening, either by promoting or repressing this process^1^. RIN, TAGL1, and FRUITFULL1 and 2 are Minichromosome Maintenance (MCM1), AGAMOUS (AG), DEFICIENS (DEF) and Serum Response Element (SRF) (MADS) domain TFs^2,3^, are highly expressed during the ripening stage and were reported as positive regulators of tomato fruit ripening^4,5^. MADS domain proteins often function as a dimer or tetramer for regulation^6^ and interaction between RIN and TAGL1 or FUL was shown by yeast-2-hybrid studies^7^. Tomato SQUAMOSA promoter-binding protein-like (SPL) transcription factor CNR is also an activator of tomato fruit ripening, involving in ethylene and lycopene biosynthesis. The spontaneous *Cnr* mutant shows a colourless pericarp with strongly reduced ethylene production^8,9^.

The tomato transcription factor AP2a is a member of the APETALA2/Ethylene Response Factor (AP2/ERF) family^10^. Using RNAi Chung *et al*.^11^ and Karlova *et al*.^12^ showed that the down regulation of *AP2a* interfered with normal ripening in fruits, including decreased carotenoid production, but increased ethylene production resulting in early onset of fruit ripening and senescence. Thus, AP2a is a negative regulator of ethylene production, but a positive regulator of other ripening aspects such as chlorophyll degradation and carotenoid biosynthesis. A negative feedback loop of *AP2a* and *CNR* during ripening was reported, in which *AP2a* was regulated by RIN, NOR and CNR, while AP2a itself negatively regulates *CNR*^12^. *AP2a* is also a target of post-transcriptional regulation by miR172^13^. NAC-NOR is a NAM, ATAF1/2 and CUC2 (NAC) domain transcription factor, containing the conserved NAC domain that functions in DNA binding as well as in dimerization with other NAC proteins^14,15^. There are 101 *NAC* genes in tomato but, only three (*NAC1*, *NAC*4 and *NAC-NOR*) were shown to be involved in regulation of fruit ripening so far^16,17^. NAC-NOR appeared to be the most strongly regulating activator based on the completely non-ripening phenotype of its spontaneous mutant^18^. In the spontaneous *nor* mutant, a 2 bp deletion in the third exon of *NAC*-*NOR* causes a frameshift, resulting in a truncated protein giving a strong non-ripening phenotype^19^. Tomato MADS domain transcription factors FUL1 and FUL2 are co-orthologs of *Arabidopsis* FRUITFULL^20^. *FUL*2 is expressed in flowers and developing green fruits, and its expression increases during ripening. *FUL1* expression is detectable in flowers, but in fruits it is much higher and specific for the ripening stage^20^. Yeast-2-hybrid protein interaction experiments showed that both could interact with RIN, which is also expressed during ripening, while FUL2 interacts with other MADS domain proteins as well^5,7^. RNAi experiments showed that *FUL1* and *FUL2* probably function redundantly in tomato fruit ripening^20–22^. Phenotypes of *FUL1/2* RNAi fruits diverged between studies, showing an orange-ripe phenotype with reduced lycopene level and relatively normal ethylene production in one study^20^, and almost green fruits with strongly reduced ethylene production in another^21,22^.

In the absence of available spontaneous mutants Virus-induced Gene Silencing (VIGS) of gene expression and RNA interference (RNAi) have often been used for evaluating gene function. Both approaches however, may suffer from incomplete suppression of expression or lack of specificity for the targeted gene. Because RNAi silencing was the most popular tool in the past decades due to the relative ease of use^23^, functional characterization of the gene of interest may have been imperfect in many cases. The action of Site-Specific Nucleases (SSN) allows targeted mutagenesis by utilizing the imperfect nature of double-strand DNA break (DSB) repair, creating mostly small INDELs which, when located in an open reading frame, can lead to frame-shifts resulting in loss-of-function alleles. Clustered Regularly Interspaced Short Palindromic Repeats (CRISPR)/ CRISPR-associated protein 9 (Cas9) has rapidly gained popularity as the SSN of choice for mutagenesis due to its high efficiency and relative ease of use. It utilizes guideRNAs (gRNAs), which recognise the target sequence to direct the endonuclease Cas9 to cut there, causing a DSB^24^. Together with efficient modular cloning strategies such as Golden Gate cloning^25^ it allows multiple gRNAs targeting more than one gene at the same time with high efficiency^26^, and CRISPR/Cas9-mutagenesis has been successfully applied in many plant species, such as *Arabidopsis*, rice, maize and tomato^27^.

Spontaneous mutants with fruit ripening phenotypes in tomato have been reported for decades and forward genetics studies have identified several of the underlying genes as encoding transcription factors, such as for the *rin* and *nor* mutants. These mutants have proven extremely valuable for both fundamental research as well as in applications. Yet, the availability of a larger set of alleles may improve our understanding of TF function further, as well as allow study of specific, true knock-out phenotypes where only RNAi studies were available before. Recently, by using CRISPR/Cas9-mediated mutagenesis *Ito et al*. illustrated that the *rin* phenotype is caused by the production of a fusion protein RIN-MC, rather than the mere loss of function of MADS-RIN^28^. After knocking out *RIN* in a wild type background, fruit ripening was affected but not blocked as it was in the original *rin* mutant^29^, and ripening was partially restored by knocking out *RIN-MC* in the *rin* background. There are so far no spontaneous mutants of *AP2a*, *FUL1* or *FUL2* reported, and RNAi or VIGS phenotypes may only partially reflect the functions of these genes.

In this study, by using CRISPR/Cas9-mutagenesis we generated genuine knock-out mutants of *AP2a*, *NAC*-*NOR*, *FUL1* and *FUL2* (as well as the latter two combined) to further study their function in tomato fruit ripening. In this way we confirm the previously found function of *AP2a*, but demonstrate that the spontaneous *nor* mutation represents a dominant-negative allele of *NAC-NOR* because a *null* allele has a milder phenotype. Moreover, true knock-out mutants of *FUL1* and *FUL2* allowed to differentiate between shared functions during ripening and a specific *FUL2* function in early fruit development.

## Results and Discussion

**Figure 1:**
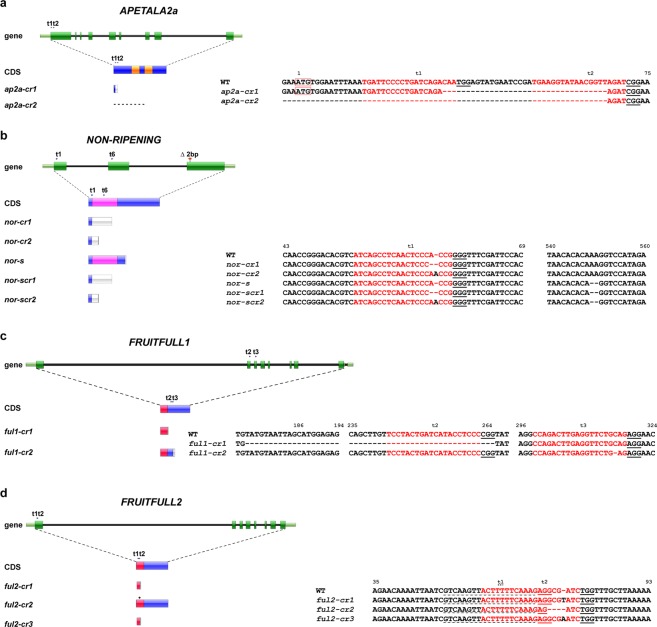
Targets for CRISPR/CAS9 mutagenesis of tomato transcription factor genes, and resulting mutant alleles. (**a**) Mutations in *AP2a*. Overview of the *AP2a* gene and protein changes in knock-out mutants. sgRNA AP2a-t1 and AP2a-t2 located in the first exon were used. (**b**) Mutations in *NAC*-*NOR*. Overview of the *NAC*-*NOR* gene and protein changes in the spontaneous *nor* (*nor-s*) and CRISPR alleles. sgRNA NOR-t1 and NOR-t6 were designed at the start and middle of the NAC domain for all mutagenesis experiments. (**c**) Mutations in *FUL1*. *FUL1* gene and protein changes in knock-out alleles. (**d**) Mutations in *FUL2*. Overview of the *FUL2* gene and protein changes in CRISPR alleles. Regions in orange, pink and red represent the AP2, NAC and MADS domain, respectively. Letters in red indicate spacer sequences and underlined are protospacer adjacent motifs (PAM). The start codon is indicated with red boxes. Numbers represent the location of the nucleotide in the coding sequence. A black diamond shows the single amino acid deletion in *ful2-cr2*.

In order to obtain knock-out mutations in the selected transcription factor genes in tomato cv. Moneyberg, we have used binary vectors containing *SpCas9* combined with 2 guideRNA-encoding expression cassettes. Two guides were used to target a single gene or, combining two guides, one for *FUL1* and *FUL2* each, to produce *ful1/ful2* double mutants. In all cases, primary transformants were genotyped for targeted mutations, selected transformants were selfed and only homozygous or biallelic mutants were used for phenotyping. The locations of the targeted sites as well as the obtained (*-cr*) alleles are shown in Fig. 1 and Supplementary Fig. 1. As can be seen in the latter, and since mutant alleles were derived from just one or two distinct guideRNAs, similar detrimental effects on the resulting protein functions were expected, and we selected those which were deemed representative for all similar mutations. Flowers were labelled at anthesis as 0 Days Post Anthesis (DPA) to record the time required to reach the Breaker (Br) stage, and ethylene production of fruits was measured at Breaker and Breaker + 5 days stages.

### *ap2a* mutants initiate fruit ripening earlier, but do not fully ripen

*AP2a* (Solyc03g044300) was reported to be a negative regulator of tomato fruit ripening initiation based on RNAi suppression of expression^12^, but true knock-out mutants were not available so far. The encoded protein of 401 amino acids contains two AP2 domains (amino acids 135 to 201 and 227 to 294), presumably involved in DNA binding^30^. By using two gRNAs, four alleles *ap2a-cr1*- *4* were obtained with deletions in the first of 9 exons (Fig. 1a and Supplementary Fig. 1a). We selected two lines with deletions most probably resulting in *null* alleles. In *ap2a-cr1* a 35 bp deletion joining the two gRNA-target sites is predicted to produce a peptide of 27 aa with no AP2 domains, while the 133 bp deletion in allele *ap2a-cr2* extends to 67 nucleotides upstream of the start codon, and therefore no AP2a protein is expected to be produced (Fig. 1a). The 133 bp deletion in *ap2a-cr2* extends into the 5′ UTR of *AP2a*, which does not necessarily affect its transcription. This mutation deletes the first start codon, as well as an alternative start codon at amino acid position 12. The next in-frame start codon at amino acid position 204 is located in exon 5 and 3′ of the first of two conserved AP2 domain-encoding regions and therefore even if used, unlikely to result in a functional protein.

**Figure 2:**
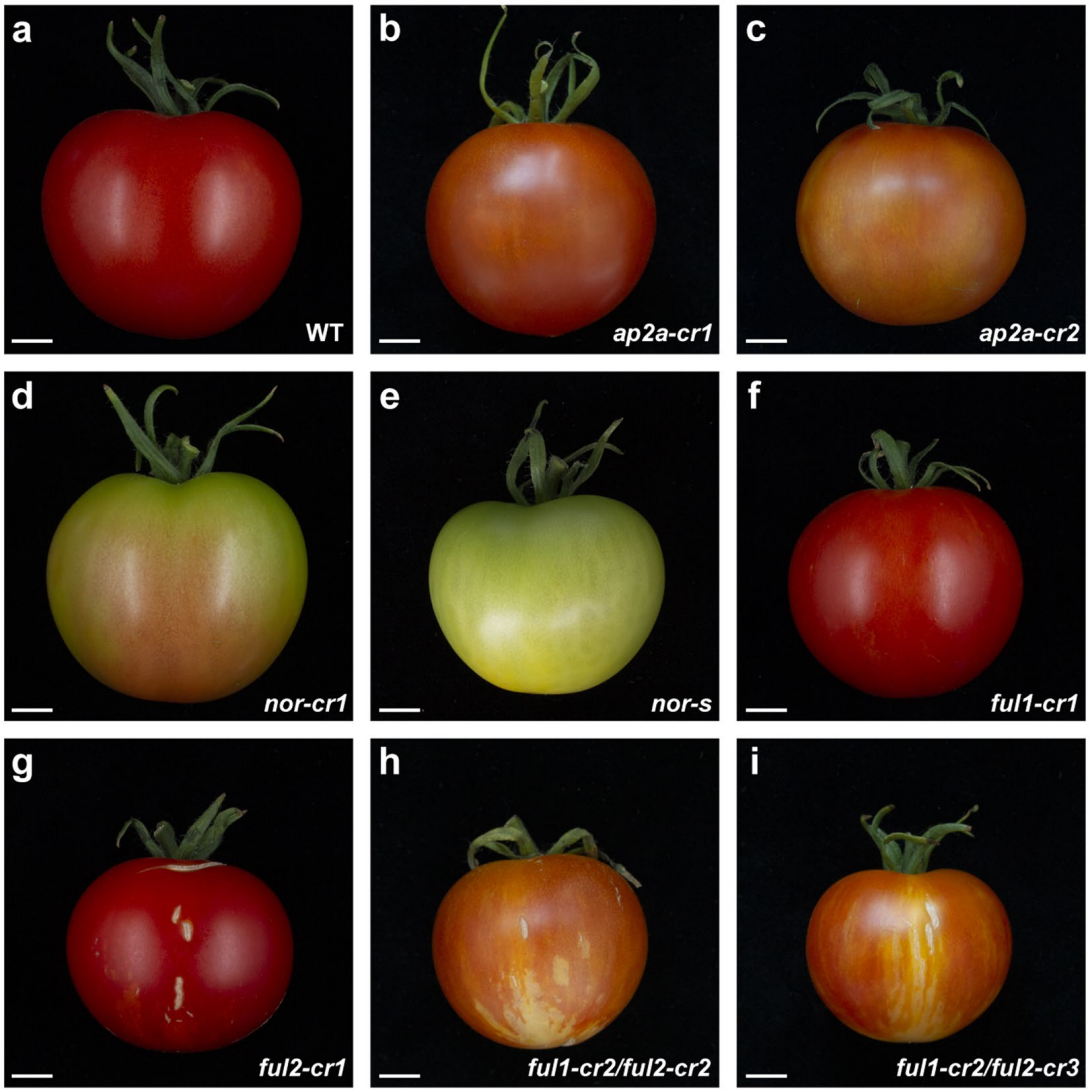
Phenotypes of mutant fruits. Fruits of homozygous mutants at 55 DPA. All mutants are in cv. Moneyberg except **(e)** which shows cv. Ailsa Craig *nor-s* for comparison at an equivalent stage. Scale bar, 1 cm.

**Figure 3:**
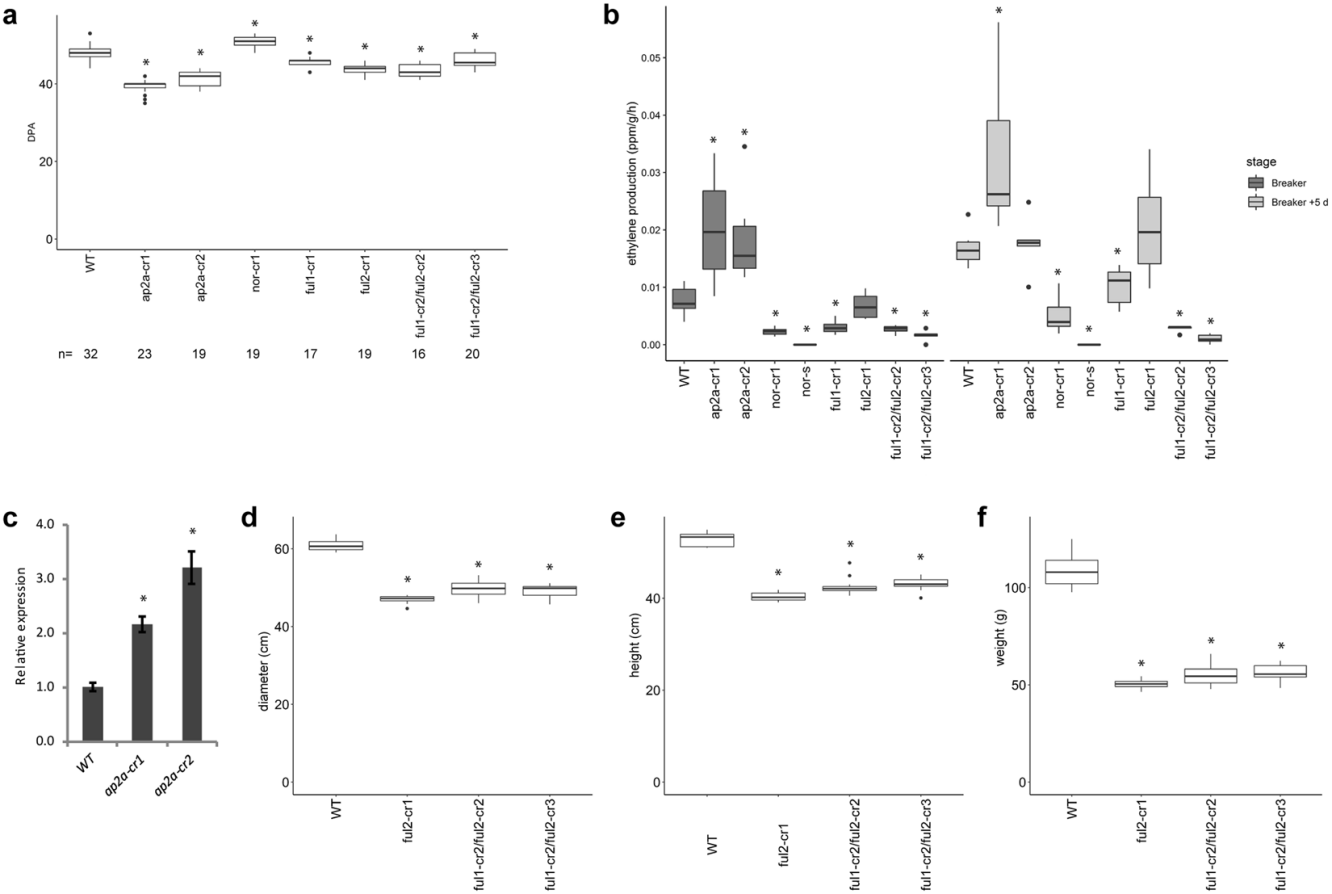
Differences in developmental and ripening processes of mutants compared to wild type. (**a**) Time to initiation of ripening (Days Post Anthesis (DPA) to Breaker) of wild type and homozygous mutants. (**b**) Ethylene production (ppm/g/h) for wild type and homozygous mutants at Br and Br+ 5 d stages and of the spontaneous *nor* mutant at the equivalent stage. Values of five or six fruits were used. (**c**) Relative *AP2a* expression in two *ap2a* knock-out lines. Error bars represent SE of means. (**d**) Diameter (cm), (**e**) Height (cm) and (**f**) Weight (**g**) of wild type and homozygous *ful2* mutant fruits. Values of eleven fruits for each genotype were used for (**d**–**f**). Asterisks show significant differences (*P* < 0.05).

When compared to wild type fruits (Fig. 2a) pericarp of the two lines remained orange/brown (Fig. 2b,c) 20 days after Breaker stage and did not become fully red (Supplementary Fig. 2a). Faster ripening was accompanied by earlier senescence: *ap2a-cr1* and *ap2a-cr2* fruits started to crack before 60 DPA while fruits from other mutants were still intact (Supplementary Fig. 2a). Fruits of the *ap2a* mutants took only 39 to 41 days to reach Br stage in *ap2a-cr1* and *ap2a-cr2*, respectively, significantly less than in wild type fruits (47 days) (Fig. 3a). This is consistent with the observed 7 days earlier colour change in RNAi fruits^11^, confirming AP2a’s negative regulatory role in the initiation of tomato fruit ripening. Fruits of both *ap2* knock-out mutants produced at least twice the wild type amount of ethylene at Br stage (Fig. 3b).

The AP2 protein in *Arabidopsis* is known to negatively regulate its own transcription by a feedback regulation^31^. While RNAi knock-down of expression usually reduces mRNA levels of the target gene with varying efficacy, remaining mRNA can still produce functional protein. Mutants producing no functional proteins, apart from giving a stronger phenotype also allow the exploration of positive or negative feedback autoregulation of the protein on its own expression. This is exemplified by expression analysis of the tomato *AP2a* mRNA in wild type and *ap2a-cr1* and *-cr2* fruits. As the mutations result in no functional protein production, mutant fruits display higher levels of *AP2a* mRNA, indicating that similar to *Arabidopsis* AP2, tomato AP2a protein negatively regulates its own transcription (Fig. 3c). The much higher ethylene production and faster ripening in *ap2a-cr1* and *ap2a-cr2* confirm AP2a’s negative role in both ethylene production as well as in initiation of tomato fruit ripening. Its positive regulatory role in ripening is shown by the orange/brown fruit colour resulting from a lack of lycopene production and a defect in chlorophyll degradation, consistent with the RNAi phenotype from Karlova *et al*.^12^.

### A *nor null* mutant has a milder phenotype than the spontaneous *nor* mutant

Ripening defects in the spontaneous *nor* mutant are likely due to a 2 bp deletion in the third exon of *NAC-NOR* (Solyc10g006880), leading to a frame shift and a truncated protein of 186 amino acid (aa) versus 355 aa for the intact gene^32^. This truncation is located after the NAC domain, leaving the possibility that the truncated protein retains its dimerization and DNA-binding capacity. To knock out *NOR* in cv. Moneyberg we designed two gRNAs for *NAC-NOR* but found no edits at position t6. Two alleles at position t1 were obtained, *nor-cr1* with a 1 bp deletion and *nor-cr3* with a 2 bp deletion 5′ of the NAC-domain coding sequence (Fig. 1b and Supplementary Fig. 1b), both resulting in a frameshift and a protein predicted to contain only 17 aa of NAC-NOR and no conserved NAC domain (Fig. 1b).

Homozygous *nor-cr1* fruits initiated ripening later than wild type fruits (Fig. 2d) by 3 days on average (time to Br stage, Fig. 3a), but surprisingly progress of ripening was only partially affected, in contrast to being totally blocked as it is in the spontaneous mutant (Fig. 2e). Homozygous *nor-cr1* exhibited an orange pericarp at 60 DPA, indicating that lycopene biosynthesis was affected. Colour change after Breaker in *nor-cr1* was much slower than in wild type fruits and the pericarp remained orange until 70 DPA and beyond (Supplementary Fig. 2b). Ethylene production in *nor-cr1* fruits at both Br and Br+ 5 d stages was significantly lower than in wild type fruits, possibly explaining the delayed initiation of ripening, but clearly higher than in spontaneous *nor* mutant fruits, where no ethylene production was detectable in the time frame where normally ripening occurs (Fig. 3b).

The *alcobaca* (*alc*) mutation, encoding a deleterious V106D substitution in the NAC domain is allelic to *nor* and displays a weaker effect on ripening^33^. By CRISPR/Cas9-induced gene replacement Yu *et al*. replaced thymine to adenine at position 317 of the *NAC-NOR* coding sequencing, creating an *alc* allele and confirming the long-shelf life character of *alc*^34^. Three Penjar accessions contain the *alc* allele, while a fourth contains an early frame-shift and a truncated protein of 6 aa, which is similar to the *nor-cr1* allele described here. Our *nor-cr1* fruits displayed delayed ripening and an orange-ripe phenotype similar to that of *alc* fruits. As the truncated protein in *nor-cr1* contains no NAC domain this likely makes *nor-cr1* a true *null* allele. A transcriptional activation region located in the C-terminal region of NAC proteins is essential for activating transcription^35^. Candidates for interaction with NAC-NOR would be NAC-NOR itself forming homodimers or the two other tomato NAC proteins involved in ripening, NAC1 and NAC4, and such an interaction was demonstrated for NAC4^16^. The decreased ethylene production in *nor-cr1* confirms that NAC-NOR, as a master regulator, is located upstream of ethylene biosynthesis and has a positive regulatory role.

### The spontaneous *nor* mutation produces a dominant-negative protein

**Figure 4:**
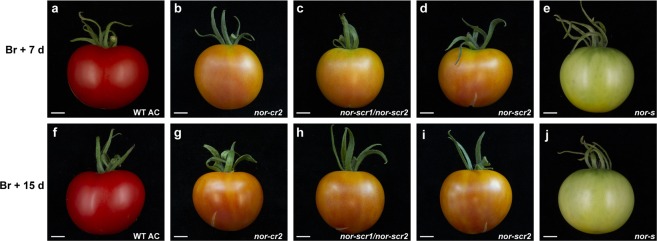
Phenotypes of homozygous mutant fruits. (**a**–**e**) Fruits at Br + 7 d and (**f**–**j**) Br + 15 d of wild type Ailsa Craig, *nor-cr2*, *nor-scr2*, *nor-scr1/nor-scr2* and the spontaneous *nor* mutant (all in cv. Ailsa Craig background) at equivalent stages. Scale bar, 1 cm.

Ripening in the spontaneous *nor* mutant, whose allele we shall from here on call *nor-s* for convenience, is totally blocked but only partially so in *nor-cr1* and *alc* fruits. Therefore we hypothesize that the non-ripening phenotype in *nor-s* is caused by the truncated protein functioning in a dominant-negative manner, where the protein product is able to interact with other NAC proteins and to bind DNA without transcriptionally activating its targets. This is reminiscent of the NAC TF SND1 in poplar, where an alternative splice variant retains the last intron and due to a premature stop forms a protein without an activation domain but with an almost intact NAC domain. This protein acts as a dominant-negative repressor of its downstream targets as well as of its own and family members’ expression^36^. This is also reminiscent of the situation with the *mads*- *rin* allele blocking ripening, although there the newly formed RIN-MC fusion protein has a novel combination of expression and transcriptional activation not seen in a *rin* knock-out line^28,37^. To further study this we introduced mutations 5′ of the location of the spontaneous mutation in *NOR* in both the spontaneous *nor* mutant as well as in the wild type Ailsa Craig background. Two alleles, *nor-scr1* and *nor-scr2* with a 1 bp deletion and insertion, respectively, were obtained in the spontaneous *nor-s* mutant background, and one allele (*nor-cr2*) with the same 1 bp insertion as *nor-scr2* was obtained in wild type Ailsa Craig (Fig. 1b). Homozygous *nor-cr2* caused an orange-ripe pericarp in Ailsa Craig fruits, and ripening in homozygous *nor-scr2* and biallelic *nor-scr1*/*nor-scr2* mutants was similar to this (Fig. 4). Therefore we can conclude that the frameshift upstream of the spontaneous mutation precludes translation of the NAC domain and negates the dominant-negative function of the spontaneous mutant, which retained an intact NAC domain.

A classical dominant- negative TF would still interact with the same regulatory DNA elements or form the same dimers as the wild type protein, but the activity of the dimer in a heterozygote would only be 25% compared to homozygous wild type thus giving a phenotype more similar to that of the homozygous mutant^38^. Heterozygous *nor-s* mutants in cv. Rutgers have an intermediate phenotype, both in timing of ripening, carotene production, as well as in ethylene production during ripening^39^. This indicates that *nor-s*’ negative effect is dose-dependent. This suggests that *nor-s* is a so-called *trans*-acting dominant-negative allele having *inter*locus interactions, rather than the classical *intra*locus interaction^38^. The tomato NAC1 and NAC4 TFs (see previous section) would be obvious candidates for such intralocus interactions.

### *FUL1* and *FUL2* have overlapping functions during fruit ripening, but *FUL2* has an additional role in fruit development

Since tomato FUL1 (Solyc06g069430) and FUL2 (Solyc03g114830) are close paralogs, it was particularly challenging to achieve RNAi-mediated knock-down of expression in a specific as well as effective manner^20^. Use of a less specific RNAi construct might have led to knock-down of multiple homologous genes. Both studies reporting knock-down of tomato *FUL* genes achieved knock-down of both concomitantly^21,40^, but only relatively weak and not completely specific knock-down of each gene individually in another study^20^. These results suggest that *FUL1* and *FUL2* were functioning at least partially redundant in tomato fruit ripening, but were inconclusive about the relative roles of the two genes. We therefore generated both *ful1* and *ful2* single mutants as well as double mutants using CRISPR/Cas9. We obtained multiple knock-out alleles in both *FUL1* and *FUL2*, alone or in combination. Double mutants were obtained with constructs containing sgRNAs for both genes. A *ful1* single mutant line containing the *ful1-cr1* allele with a 91 bp deletion, presumably caused by microhomology-directed repair of the double strand break, which completely deleted the second exon, produces a truncated protein of 62 aa consisting of the MADS domain only (Fig. 1c). The 1 bp deletion in mutant allele *ful1-cr2* leads to a truncation at amino acid position 105 (Fig. 1c). Both alleles disable FUL1 function. In the *ful2* single mutant, the *ful2-cr1* allele has a 1 bp insertion in the middle of the MADS domain-encoding region resulting in a truncated protein with only 29 aa (Fig. 1d). A 3 bp deletion in *FUL2* in the *ful2-cr2* allele allows production of the entire protein minus one amino acid, arginine 25 in the middle of the extremely conserved MADS domain. This mutation is very likely to be deleterious to protein function, as further supported by analysis in the Provean protein website (Provean score: −12.037)^41^. Other obtained alleles are shown in Supplementary Fig. 1c,d. We also checked the sequences of the corresponding parts in the non-target paralog for *FUL1* and *FUL2* (Supplementary Fig. 1c,d) reciprocally but found no mutations there, demonstrating the high specificity of sgRNAs.

There were no apparent differences in final overall fruit colour between *ful1-cr1*, *ful2-cr1* and wild type fruits at 55 DPA (Fig. 2a,f,g). However, in both double mutants (Fig. 2h,i) the pericarp stayed orange until 60 DPA (Supplementary Fig. 2a) and did not reach a red ripe colour as in the wild type fruits (Fig. 2a and Supplementary Fig. 2a), which is similar to the phenotype observed for the RNAi *FUL1*/*FUL2* silenced lines in the study of Bemer *et al*.^20^.

**Figure 5:**
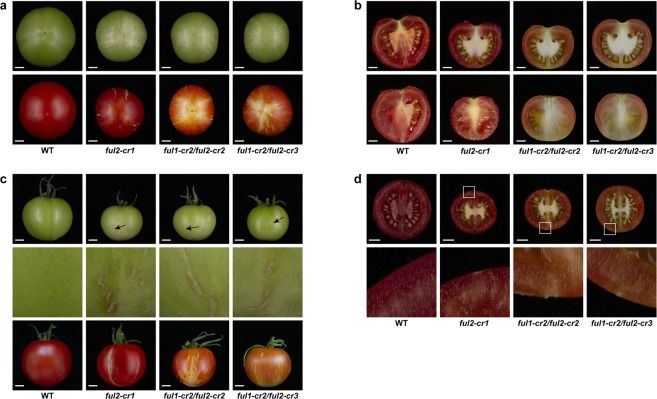
Details of fruit phenotypes and differences in *ful2* mutants compared to wild type. (**a**) Bottom view of *ful2* single and double mutants. Light coloured stripes only at the bottom of *ful2* mutant fruits appear from early green fruit stage. (**b**) Longitudinally sliced fruits of *ful2 null* mutants with light coloured-pericarp at the bottom of fruits. (**c**) Details of cracks in *ful2* mutants. Cracks in all *ful2* mutants at unripe (top), including details (middle) and ripe (bottom) stage. Black arrows indicate cracks in unripe fruits. (**d**) Latitudinally sliced fruits of *ful2* mutants. Boxed regions are enlarged in the lower row. Scale bar, 1 cm.

Interestingly, we observed a phenotype in *ful2-cr* mutants that had not been described before in the RNAi knock-down lines. In early stages of fruit development, superficial stripes lighter than the surrounding pericarp were visible at the bottom of homozygous *ful2-cr1* fruits, but not in homozygous *ful1-cr1* fruits, which became less distinguishable as fruits ripened (Figs 2g, 5a and Supplementary Fig. 2c). Significantly lighter-pigmented stripes were also found at the bottom of all fruits in the two double mutants *ful1-cr2/ful2-cr2* and *ful1-cr2/ful2-cr3*, which only changed from white to yellow around the time when wild type fruits are fully ripe (Figs 2h,i and 5a). These stripes were not only visible superficially, but also in the mesocarp of sections of both homozygous *ful1/ful2* double mutants, making the pericarp at the bottom region and septum much lighter coloured than the rest of the fruit (Fig. 5b). Moreover, vertical, possibly suberized cracks in the surface of all *ful2* mutant lines, including double mutant lines, were visible from the early stages of fruit development (Figs 2g–i and 5c,d). Additionally, the columella and placenta of *ful2* and *ful1/ful2* fruits remained white when fruits were fully ripe (Fig. 5b,d). Besides, we noticed that fruits of all the mutants with a *ful2 null* allele were more than 10 mm smaller than wild type fruits, both in diameter and in height (Fig. 3d,e) and almost half the weight of wild type fruits (Fig. 3f). Also there was a significant effect of *ful2* on time to onset of ripening (Br stage). It took 46 days in *ful1-cr1* and only 43 days from anthesis to Breaker in *ful2-cr1* fruits, significantly less than in wild type fruits (Fig. 3a and Supplementary Fig. 2a). The time to ripening was also significantly shorter than in wild type fruits in both double mutant lines (Fig. 3a), but the pericarp stayed orange until 60 DPA and did not reach a red ripe colour as in the wild type fruits. We speculate that the smaller fruit size of *ful2* mutants may have a causal relation to the earlier ripening phenotype, but this will require more study.

We also measured ethylene production in all mutants, and found significantly lower production in the *ful1-cr1* single mutants and the double mutants, but not so in the *ful2-cr1* mutants (Fig. 3b). The strong reduction in the *ful1-cr* mutant indicates that *FUL1* is an important regulator of ethylene biosynthesis in the ripening phase, although its reduction in the single mutant has little effect on visible ripening aspects. However, *FUL2* also contributes, because ethylene production is even more reduced in the *ful1-cr2/ful2-cr3* double mutants. Compared to wild type fruits, ethylene production decreased to 32% and 17% in two double mutant lines at Breaker stage, and to only 15% and 5% at Br+ 5 d (Fig. 3b). Thus, although *FUL1* and *FUL2* were reported to act redundantly during fruit ripening, *ful2* mutants affect unique aspects of fruit development, both time to ripening and as well as fruit skin integrity, which are similarly affected in double mutants. By contrast, *ful1-cr1* showed a very mild difference from WT without cracks or stripes in the pericarp, nor a difference in time to ripening, but a stronger effect on ethylene production. This is consistent with the strong increase in *FUL1* expression during ripening, while *FUL2* is expressed in developing green fruits as well. Some of the differences in the functions of *FUL1* and *FUL2* that have become apparent in this study may also reflect tissue-specific and temporal differences in expression. According to the Tomato Expression Atlas (TEA)^42^ *FUL2* expression is especially high in internal fruit tissues (columella, placenta, locule tissues) while *FUL1* expression, during ripening, is more evenly distributed over pericarp and internal tissues (Supplementary Fig. 3). *FUL2* expression however is higher than *FUL1* expression in all fruit tissues up to the mature green stage.

Phenotypes of *ful1* or *ful2 null* alleles are reported for the first time in our study and although their combined mutations had a severe ripening phenotype, they are very different from *FUL1/FUL2* double knock-down lines reported by Shima *et al*. or Wang *et al*., in which they showed a totally blocked ripening phenotype^21^ or bright yellow fruits^40^ when silencing both *FUL1* and *FUL2*. The orange pericarp of *ful1/ful2* true knock-out mutants is similar to what Bemer *et al*.^20^ presented in their RNAi experiment, while single mutants exhibited normal red-ripe pericarp, confirming their redundant function in fruit ripening. Light coloured stripes at the bottom region and vertical (possibly suberized) cracks in the surface of all lines containing homozygous *ful2* alleles have never been reported before. They are visible from the green fruit stage, illustrating additional roles of *FUL2* in early fruit development and in carotenoid biosynthesis. Fujisawa *et al*. showed that the promoter of *PHYTOENE SYNTHASE 1* (*PSY1*) was a direct target of RIN and FUL1, but not of FUL2^22^, suggesting that another mechanism is responsible for FUL2 regulating fruit pigmentation. Possibly FUL2 is involved in chloroplast formation, stability, or function during early fruit development, which would account for the lighter green areas. The cracks in the surface of all *ful2* (both single and double mutant) lines are a unique phenotype of *ful2* mutants that is not shared with *ful1* single mutants. The aforementioned RNAi studies of combined *FUL1*/*FUL2*-function all reported thinner-cuticles, leading to faster water loss in ripe fruits^20,40^. The observed cracks in the *ful2* CRISPR mutants indicate that this may well represent a specific *FUL2* function in cutin formation or epidermal development in tomato fruit.

The additive effect in ethylene production among single and double mutants proves the partially redundant functions of *FUL1* and *FUL2* in ethylene biosynthesis and fruit ripening. In contrast to the unchanged ethylene production in knock-down lines shown by Bemer *et al*.^20^, the strongly decreased ethylene level in *ful1*/*2* double knock-out mutants in our study illustrates that *FUL1* and *FUL2* regulate tomato fruit ripening via ethylene biosynthesis, consistent with other studies^21,40^. These discrepancies between studies are possibly caused by the use of different genotypes (MicroTom, Ailsa Craig or Moneyberg), and by the limited downregulation in the RNAi lines previously analysed^12^. Here it is shown that only one third or less of wild type ethylene production in *ful1/ful2* double mutants could still support some ripening progression, as it does in the *nor-cr* mutants indicating that the initiation of ripening may only require a limited amount of ethylene, and once it reaches a threshold, ripening starts even if not progressing to its full extent.

In conclusion, we have demonstrated the utility of CRISPR/Cas9-mutagenesis in tomato for reassessing transcription factor gene functions. Some phenotypes closely resembled those that were previously reported, but in addition allow the study of regulatory features such as an auto feedback regulation of transcription (*ap2a*) or complementation by retransformation. For others, studying alternative alleles gives more insight into gene function by revealing a distinction between *null* and dominant-negative alleles (*nor-s*), respectively. Finally, gene-specific mutations allow the separation of functions (or demonstrate redundancy) of pairs of very similar paralogs (*ful1* and *ful2*), which are difficult to separate by older methods such as RNAi or VIGS.

## Materials and Methods

### Plant materials and growing conditions

Tomato cv. Moneyberg, Ailsa Craig (AC) and the AC *nor* mutant (the latter two obtained from the Tomato Genetics Resource Centre, TGRC) were used for the *Agrobacterium tumefaciens*-mediated transformation experiments^43^. Tissue culture was done in a growth chamber with 16 h light and 8 h dark at 25 °C. Larger plants before flowering were moved to the greenhouse facilities of Unifarm, Wageningen University & Research and grown and phenotyped under standard greenhouse conditions.

### gRNA design and mutagenesis constructs

Online programs CRISPR-P 1.0 (http://crispr.hzau.edu.cn/CRISPR/)^44^ and CRISPOR (http://crispor.tefor.net/crispor.py)^45^ were used for designing gRNAs and for excluding off-targets.

The MoClo Toolkit^46^ was used to assemble constructs with gRNAs targeting each gene and the Golden Gate cloning strategy was used as described earlier to assemble binary vectors for tomato mutagenesis^47^. Briefly, each gRNA fused to the synthetic Arabidopsis U6 promoter as AtU6p::gRNA was ligated in a Level 1 vector. Level 1 constructs *pICH47732-NOSpro::NPTII::OCST*, *pICH47742-35S::Cas9::NOST*, *pICH47732-*gRNA1, *pICH47742-*gRNA2 and the linker *pICH41780* were cut/ligated into the Level 2 vector *pICSL4723* as described^48^. All primers used for amplifying gRNAs with backbones are listed in Supplementary Table 1.

### Transgenic plant genotyping

Genomic DNA from young leaves was isolated using the CTAB method^49^. PCRs for *Cas9* and *NPT2* were performed for all regenerated plants and only from the *Cas9/NPT2* positive plants target regions were sequenced. Heterozygous and biallelic mutants were selfed and T_1_ seedlings were screened for the absence of *Cas9* and the presences of homozygous mutations. Homozygous mutants without *Cas9* after segregation were used for further study. All primers used for genotyping are listed in Supplementary Table 1.

### Fruit development phenotyping

Two plants per genotype were used for phenotyping. Tomato flowers of all the mutant lines and WT were vibrated and labelled at the day when they were first fully open as 0 Days Post Anthesis (DPA). Data from at least fifteen flowers/fruits per genotype was used for calculating the time taken to reach Breaker stage. Fruits at 35, 40, 45, 50, 55 and 60 DPA were collected for photography. Ten or eleven fruits at 60 DPA were collected for size and weight measurement. Normal distribution of data was confirmed with the R package version 3.5.0 and ANOVA was used to test for the significance of differences between individual mutants and wild type, respectively.

### Ethylene measurements

Tomato fruits at Breaker stage and Breaker + 5 d were harvested. After being in open air for 30 min at room temperature fruits were placed in sealed jars for 3 h. Ethylene concentration was measured when immediately after sealing and after 3 h by injecting 1.5 mL gas to a Focus GC gas chromatograph. Ethylene production was calculated as ppm per gram of fruit per hour (ppm/g/h). Values of five or six fruits per genotype were used for analysis. Since ethylene data did not display a normal distribution, generalized linear model regression with a quasibinomial model was used to determine the significance of differences between genotypes.

### Real-time PCR gene expression analysis

RNA was isolated from pericarp of fruits at Br+ 5 d stage by using the InviTrap Spin Plant RNA kit (Stratec) and cDNA was synthesized by the iScript cDNA synthesis kit (Bio-Rad). Two replicates were analysed per plant containing one fruit each from two plants per line. Primers used for qRT-PCR are listed in Supplementary Table 1. iQ SYBR Green Supermix (Bio-Rad) and iCycler iQ5 system (Bio-Rad) were used for quantitative RT PCR. *Actin* was used as a reference and relative expression changes of *AP2a* were calculated according to 2^−ΔΔCt^ method as described^50^. Student’s t-test was performed to detect significant differences.

## Supplementary information

Supplementary figures & information

## Data Availability

All data in this study are available in the article and supplementary files, or from the corresponding author upon request. Sequence data can be found in the Sol Genomics Network with the following accession numbers: *AP2a* (Solyc03g044300 (https://solgenomics.net/locus/17739/view)), *NAC-NOR* (Solyc10g006880 (https://solgenomics.net/locus/1031/view)), *FUL1* (Solyc06g069430 (https://solgenomics.net/locus/581/view)) and *FUL2* (Solyc03g114830 (https://solgenomics.net/locus/582/view)).
