# Supplementary material for: Re-evaluation of transcription factor function in tomato fruit development and ripening with CRISPR/Cas9-mutagenesis
Source: Sci Rep. 2019 Feb 8;9:1696. doi: 10.1038/s41598-018-38170-6 (PMC6368595; doi:10.1038/s41598-018-38170-6)
Supplement: Supplementary file 1 — Supplementary figures & information [file 41598_2018_38170_MOESM1_ESM.pdf]

Re-evaluation of transcription factor function in tomato fruit development and ripening with CRISPR/Cas9-mutagenesis

Rufang Wang[1,2], Eveline Carla da Rocha Tavano[3], Michiel Lammers[2], Adriana Pinheiro Martinelli[3], Gerco C. Angenent[1,2], and Ruud A. de Maagd[2*]

[1]Laboratory of Molecular Biology, Wageningen University, The Netherlands

[2]Bioscience, Wageningen Plant Research, The Netherlands

[3]CENA, University of Sao Paulo, Piracicaba, Brazil

*Corresponding author. Email: ruud.demaagd@wur.nl

a

```
               -69       -60   -5   1                     t1                                    t2             74
WT             ACATCAATAT   AAGAAATGTGGAATTTAAATGATTCCCCTGATCAGACAATGGAGTATGAATCCGATGAAGGTATAACGGTTAGATCGGA
ap2a-cr1       ACATCAATAT   AAGAAATGTGGAATTTAAATGATTCCCCTGATCAGA-----------------------------------AGATCGGA
ap2a-cr2       AC--------   -----------------------------------------------------------------------AGATCGGA
ap2a-cr3       ACATCAATAT   AAGAAATGTGGAATTTAAATGATTCCCCTGATCAGA------------------------------------GATCGGA
ap2a-cr4       ACATCAATAT   AAGAAATGTGGAATTTAAATGATTCCCCTGATCAGACAATGGAGTATGAATCCGATGAAGGTATAACGGT-AGATCGGA
```

b

```
               43                t1                 69   540            560
WT             CAACCGGGACACGTCATCAGCCTCAACTCCC-ACCGGGGTTTCGATTCCAC   TAACACACAAAGGTCCATAGA
nor-cr1        CAACCGGGACACGTCATCAGCCTCAACTCCC--CCGGGGTTTCGATTCCAC   TAACACACAAAGGTCCATAGA
nor-cr2        CAACCGGGACACGTCATCAGCCTCAACTCCCAACCGGGGTTTCGATTCCAC   TAACACACAAAGGTCCATAGA
nor-cr3        CAACCGGGACACGTCATCAGCCTCAACTC---ACCGGGGTTTCGATTCCAC   TAACACACAAAGGTCCATAGA
nor-s          CAACCGGGACACGTCATCAGCCTCAACTCCC-ACCGGGGTTTCGATTCCAC   TAACACACA--GGTCCATAGA
nor-scr1       CAACCGGGACACGTCATCAGCCTCAACTCCC--CCGGGGTTTCGATTCCAC   TAACACACA--GGTCCATAGA
nor-scr2       CAACCGGGACACGTCATCAGCCTCAACTCCCAACCGGGGTTTCGATTCCAC   TAACACACA--GGTCCATAGA
```

c

```
                              186    195 234           t2          264   291            t3             324
WT             ATGTATGTAATTAGCATGGAGAGG   ACAGCTTGTTCCTACTGATCATACCTCCCCGGTAT   ACTTAAGGCCAGACTTGAGGTTCTG-CAGAGGAAC
full-cr1       ATG---------------------   --------------------------------TAT   ACTTAAGGCCAGACTTGAGGTTCTG-CAGAGGAAC
full-cr2       ATGTATGTAATTAGCATGGAGAGG   ACAGCTTGTTCCTACTGATCATACCTCCCCGGTAT   ACTTAAGGCCAGACTTGAGGTTCTG--AGAGGAAC
full-cr3       ATGTATGTAATTAGCATGGAGAGG   ACAGCTTGTTCCTACTGATCATACCTCCCCGGTAT   ACTTAAGGCCAGACTTGAGGTTC---CAGAGGAAC
full-cr4       ATGTATGTAATTAGCATGGAGAGG   ACAGCTTGTTCCTACTGATCATAC--CCCCGGTAT   ACTTAAGGCCAGACTTGAGGTTCTG-CAGAGGAAC
full-cr5       ATGTATGTAATTAGCATGGAGAGG   ACAGCTTGTTCCTACTGAT-------CCCCGGTAT   ACTTAAGGCCAGACTTGAGGTTCTG-CAGAGGAAC
full-cr6       ATGTATGTAATTAGCATGGAGAGG   ACAGCTTGTTCCTACTGATCA-----CCCCGGTAT   ACTTAAGGCCAGACTTGAGGTTCTG-CAGAGGAAC
full-cr7       ATGTATGTAATTAGCATGGAGAGG   ACAGCTTGTTCCTACTGATCATACCTCCCCGGTAT   ACTTAAGGCCAGACTTGAGGTTCT--CAGAGGAAC
full-cr8       ATGTATGTAATTAGCATGGAGAGG   ACAGCTTGTTCCTACTGATCATACCTCCCCGGTAT   ACTTAAGGCCAGACTTGAGGTTCTGACAGAGGAAC
full-cr9       ATGTATGTAATTAGCATGGAGAGG   ACAGCTTGTTCCTACTGATCATACCTCCCCGGTAT   ACTTAAGGCCAGACTTGAGGTT----CAGAGGAAC
full-cr10      ATGTATGTAATTAGCATGGAGAGG   ACAGCTTGTTCCTACTGATCATA---CCCCGGTAT   ACTTAAGGCCAGACTTGAGGTTCTG-CAGAGGAAC
full-cr11      ATGTATGTAATTAGCATGGAGAGG   ACAGCTTGTTCCTACTGATC------CCCCGGTAT   ACTTAAGGCCAGACTTGAGGTTCTG-CAGAGGAAC
full-cr12      ATGTATGTAATTAGCATGGAGAGG   ACAGCTTGTTCCTACTGAT--------------AT   ACTTAAGGCCAGACTTGAGGTTCTG-CAGAGGAAC
full-cr13      ATGTATGTAATTAGCATGGAGAGG   ACAGCTTGTTCCTACTGATCATACCTCCCCGGTAT   ACTTAAGGCCAGACTTGAGGTTCTGCCAGAGGAAC
full-cr14      ATGTATGTAATTAGCATGGAGAGG   ACAGCTTGTTCCTACTGATCATA---CCCCGGTAT   ACTTAAGGCCAGACTTGAGGTTCT--CAGAGGAAC
full-cr15      ATGTATGTAATTAGCATGGAGAGG   ACAGCTTGTTCCTACTGATCATACCTCCCCGGTAT   ACTTAAGGCCAGACTTGAGGTTCTGTCAGAGGAAC
FUL2           TCTTGTCATGTTAGCATGGAAAGG   GCAGCTTAATGCTACTGATATTATAACCCCGGTAT   GCTTAAGGCCAGACTTGAGGTTTTG-CAAAGAAAC
```

d

```
               35                  t1          t2                   93
WT             AGAACAAAATTAATCGTCAAGTTACTTTTTCA-AAGAGGCG-ATCTGGTTTGCTTAAAAA
ful2-cr1       AGAACAAAATTAATCGTCAAGTTACTTTTTCA-AAGAGGCGTATCTGGTTTGCTTAAAAA
ful2-cr2       AGAACAAAATTAATCGTCAAGTTACTTTTTCA-AAGAG----ATCTGGTTTGCTTAAAAA
ful2-cr3       AGAACAAAATTAATCGTCAAGTTACTTTTTCA-AAGAGGCGAATCTGGTTTGCTTAAAAA
ful2-cr4       AGAACAAAATTAATCGTCAAGTTACTTTTTCAAAAGAGGCG-ATCTGGTTTGCTTAAAAA
ful2-cr5       AGAACAAAATTAATCGTCAAGTTACTTTTTCA-AAGAGGC--ATCTGGTTTGCTTAAAAA
FUL1           AGAACAAAATTAACCGTCAAGTTACCTTCTCG-AAACGTCG-ATCTGGTTTGCTGAAGAA
```

Supplementary Figure 1. All alleles obtained by using CRISPR/CAS9 mutagenesis. (**a**) All mutant alleles in *AP2a*. (**b**) All alleles obtained in *NAC-NOR*. (**c**) 15 alleles obtained in *FUL1*. Alignment differences between *FUL1* and the corresponding region in *FUL2* are shown in green. (**d**) Mutant alleles in *FUL2*. Alignment differences between *FUL2* and the corresponding region in *FUL1* are shown in green. Letters in red indicate spacer sequences and underlined are protospacer adjacent motifs (PAM). The start codon is indicated with red boxes. Numbers represent the location of the nucleotide in coding sequence.

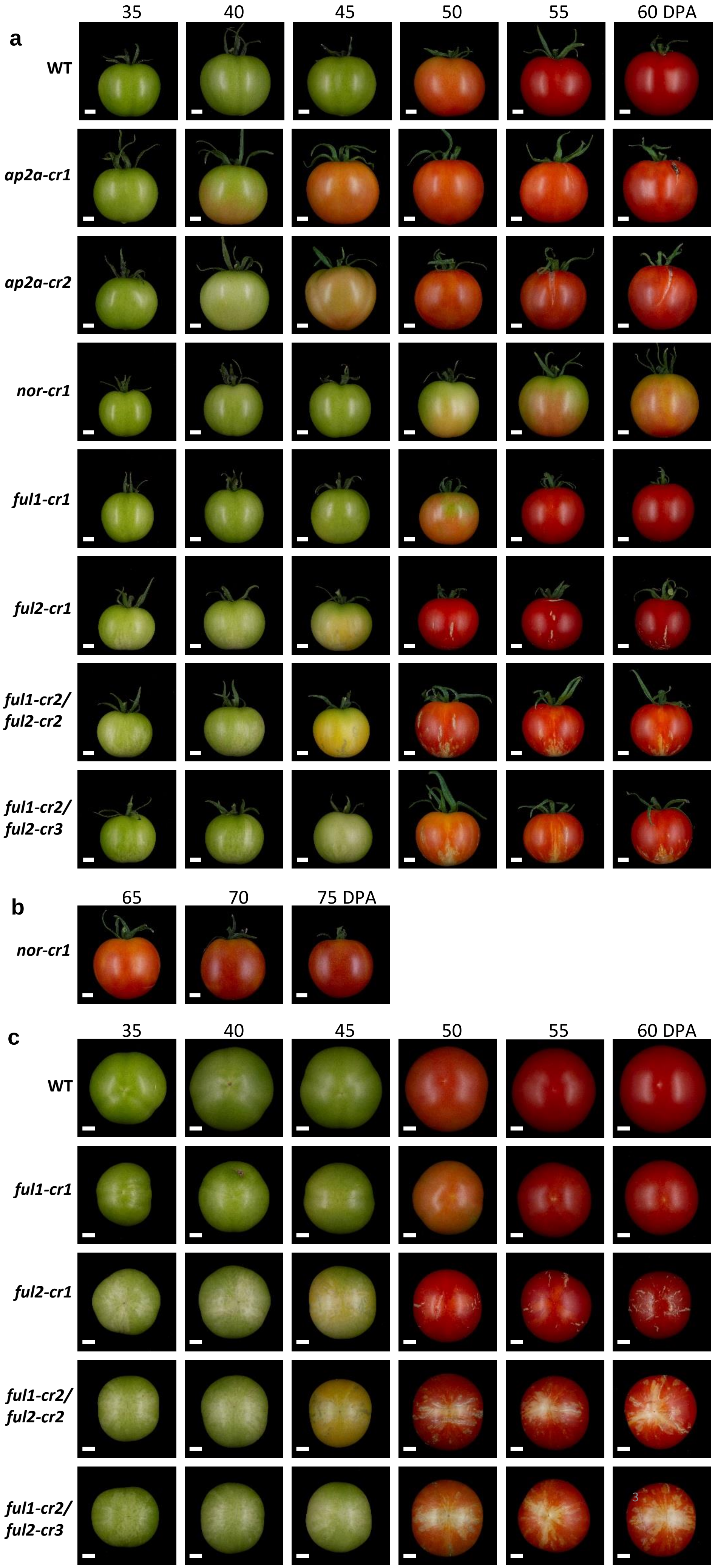

Supplementary Figure 2. Development and ripening of homozygous mutants in the T_1 generation. (**a**) Pictures taken every 5 days from 35 DPA show the difference in developmental and ripening processes among all lines in this study. (**b**) Ripening in fruits of *nor-cr1* is slower than other mutants and its pericarp still stays in dark orange until 75 DPA. (**c**) Development of stripes at the bottom of all *ful* mutants. Light coloured-stripes are visible from the early stages of fruit development and only in mutants with *ful2* alleles. Scale bar, 1 cm.

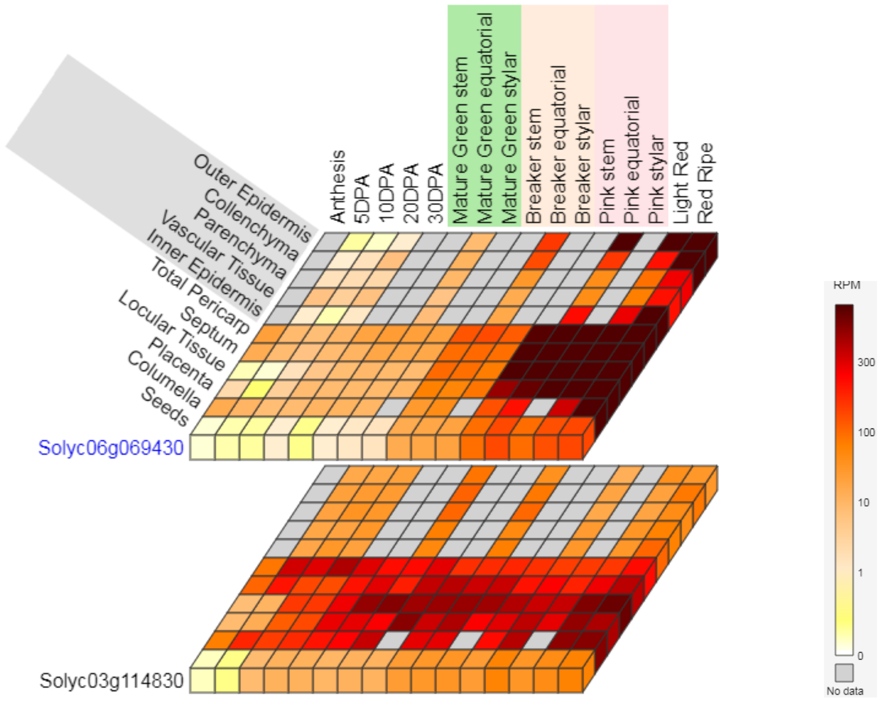

Supplementary Figure 3. Expression of *FUL1* (Solyc06g069430) and *FUL2* (Solyc03g114830) in different tissues and stages of tomato fruit development and ripening from the TEA.

Primer name	Sequence (5'-3')	Description
AP2a-t1-F	TGTGGTCTCAATTGGATTCCCCTGATCAGACAAGTTTTAGAG CTAGAAATAGCAAG	Forward primer for target 1 in *AP2a*
AP2a-t2-F	TGTGGTCTCAATTGGAAGGTATAACGGTTAGATGTTTTAGAG CTAGAAATAGCAAG	Forward primer for target 2 in *AP2a*
NOR-t1-F	TGTGGTCTCAATTGATCAGCCTCAACTCCCACCGGTTTTAGA GCTAGAAATAGCAAG	Forward primer for target 1 in *NOR*
NOR-t6-F	TGTGGTCTCAATTGGAAAATATCCTAACGGGGCGGTTTTAGA GCTAGAAATAGCAAG	Forward primer for target 6 in *NOR*
FUL1-t2-F	TGTGGTCTCAATTGCCTACTGATCATACCTCCCGTTTTAGAGC TAGAAATAGCAAG	Forward primer for target 2 in *FUL1*
FUL1-t3-F	TGTGGTCTCAATTGCAGACTTGAGGTTCTGCAGGTTTTAGAG CTAGAAATAGCAAG	Forward primer for target 3 in *FUL1*
FUL2-t1-F	TGTGGTCTCAATTGTCAAGTTACTTTTTCAAAGGTTTTAGAGC TAGAAATAGCAAG	Forward primer for target 1 in *FUL2*
FUL2-t2-F	TGTGGTCTCAATTGCTTTTTCAAAGAGGCGATCGTTTTAGAGC TAGAAATAGCAAG	Forward primer for target 2 in *FUL2*
CRISPR Universal R	TGTGGTCTCAAGCGTAATGCCAACTTTGTAC	Universal Reverse primer for all gRNAs
NPT2-F	AGACAATCGGCTGCTCTGAT	Genotyping transgenic plants for NPT2
NPT2-R	AGCCAACGCTATGTCCTGAT	Genotyping transgenic plants for NPT2
CAS9-F	CTTTGGCAATATCGTGGACG	Genotyping transgenic plants for CAS9
CAS9-R	CGTTCTTCTTCTCCCCAGGG	Genotyping transgenic plants for CAS9
AP2a-F	CGTGGGGGTGTATTTAACG	Genotyping *ap2a* mutants
AP2a-R	GGCGATTCCAAATTGTGG	Genotyping *ap2a* mutants
NOR-F	CGAATTTATCAACCTCGTA	Genotyping *nor* mutants
NOR-R	TTATCACAACCAAGTGGC	Genotyping *nor* mutants
FUL1-F	GACCTTCGCTTATAGCTCTATCCC	Genotyping *ful1* mutants
FUL1-R	CTTCTCCCACATAATGCCTGC	Genotyping *ful1* mutants
FUL2IN1-F	TTTCTTCCGTCTGTCTCCA	Check FUL2 targets region in *ful1* mutants
FUL2IN1-R	GAATCAGGGCGGCTCAATA	Check FUL2 targets region in *ful1* mutants
FUL2-F	CTGGGGAGATCCTTCC	Genotyping *ful2* mutants
FUL2-R	TGAGTCCAACTTCAGCATCG	Genotyping *ful2* mutants
FUL1IN2-F	CGCTGGCTTAGTGCAAGTA	Check FUL1 targets region in *ful2* mutants
FUL1IN2-R	AGCAGAATCGAGCTGGTGT	Check FUL1 targets region in *ful2* mutants
Actin-F	TGAGAGGTGCCTGATGCATTGC	qPCR for *Actin*
Actin-R	ACGCTTCGACCAAGGGATGG	qPCR for *Actin*
qAP2a-F	AACGGACCACAATCTTGAC	qPCR for *AP2a*
qAP2a-R	CTGCTCGGAGTCTGAACC	qPCR for *AP2a*

Supplementary Table 1. Primers used in this study.
